# An extended version of Necessary Condition Analysis (NCA) allows more specific conclusions: an example involving well-being and resilience

**DOI:** 10.1186/s12888-022-03774-w

**Published:** 2022-02-15

**Authors:** Kimmo Sorjonen, Bo Melin

**Affiliations:** Division of Psychology, Department of Clinical Neuroscience, Karolinska Institutet, 171 77 Stockholm, Sweden

**Keywords:** Extended version, Necessary condition analysis (NCA), Necessity, Re-analysis of data, Resilience, Sufficiency, Well-being

## Abstract

**Background:**

After conducting necessary condition analysis (NCA), researchers have concluded that a certain, not too low, level of well-being is necessary but not sufficient for a high level of resilience. However, as acknowledged by the developers of the test, NCA only evaluates if the association between two variables is characterized by some unspecified type of non-randomness and not conditions of necessity.

**Method:**

Earlier reported data on the association between well-being and resilience among Filipino adults (*N* = 533) in COVID-19 quarantine were re-analyzed with an extended version of NCA.

**Results:**

Analyses indicated a significant necessity effect of resilience on overall well-being, which is not logically compatible with well-being being necessary but not sufficient for resilience. Analyses with an extended version of NCA suggested that the association between overall well-being and resilience was characterized by equal degrees of necessity and sufficiency.

**Conclusions:**

The original version of NCA is only capable of evaluating if the association between two variables is characterized by some unspecified type of non-randomness. The extended version of NCA allows researchers to draw more specific conclusions.

## Introduction

According to rules of logic, if a condition X is necessary but not sufficient for another condition Y, there are no instances where X is absent and Y is present, but there are instances where X is present and Y is absent. Consequently, as there are instances where X is present and Y is absent, if X is necessary but not sufficient for Y, Y cannot, at the same time, be necessary for X. However, as there are no instances where X is absent and Y is present, if X is necessary but not sufficient for Y, Y must, at the same time, be sufficient for X.

Necessary condition analysis (NCA) is a method that was originally developed to help researchers identify conditions that are necessary but not sufficient for some outcome of interest [1]. According to the logic of the test, the size of the empty space in the upper-left corner when plotting two variables, X and Y, against each other indicates to what degree a low value on X precludes a high value on Y, i.e. to what degree X is necessary for Y [1]. Subsequently, NCA has been extended with a significance test that employs permutations [2]. Researchers employing NCA have concluded, for example, that a certain minimum level of safety consciousness is required to achieve top productivity results among long-haul truck drivers [3], that a certain degree of workplace spirituality is necessary for high levels of employee commitment, job satisfaction, and work-life balance satisfaction [4], and that a not too low level of intelligence is necessary for creativity [5–7].

Well-being and resilience seem to be associated [8, 9]. It has been suggested that this association is due to positive emotions, e.g. well-being, facilitating resilience [10, 11]. However, it has also been proposed that resilience may promote positive emotions [12–15]. After conducting analyses with NCA, Camitan and Bajin concluded that well-being is necessary but not sufficient for resilience [16].

However, there are mounting evidence that NCA does not correctly identify conditions that are necessary but not sufficient for the outcome. Sorjonen and Melin [17, 18] have shown that if X is sufficient for Y, X does not need to be necessary for Y for a significant result in NCA, which is in exact contradiction to the originally stated objective of NCA (see also [19]). The developers of NCA have acknowledged that NCA does not assess if X can be assumed to be necessary for Y, and even less if X can be assumed to be necessary but not sufficient for Y. Instead, NCA has been described as a null hypothesis test and a significant finding only indicates that the association between X and Y is characterized by some unspecified type of non-randomness [20]. However, NCA seems inferior to ordinary linear regression analysis for detecting non-random associations [21].

In order to increase the specificity and usefulness of results obtained through NCA, Sorjonen and Melin [18] have proposed an extended version of NCA, where both the degree of necessity and the degree of sufficiency, as well as the difference between these two, are calculated. Degree of sufficiency is operationalized as the size of the empty space in the lower-right corner when plotting the two variables against each other (see the Result section for an illustration). This sufficiency effect of X on Y equals the necessity effect of Y on X. The significance of the difference between degree of necessity and degree of sufficiency can be evaluated through bootstrapping and a significant finding indicates that the association between X and Y is characterized either by a higher degree of necessity than sufficiency (significant positive difference) or by a higher degree of sufficiency than necessity (significant negative difference). A non-significant difference suggests equal degrees of necessity and sufficiency. However, Sorjonen and Melin warn against drawing conclusions about a significant difference unless the stronger effect, either necessity or sufficiency, has been indicated as significant by the permutation test in NCA [18].

Given the limitations of NCA, the objective of the present study was to evaluate if well-being really can be assumed to be necessary but not sufficient for resilience by re-analyzing the data used by Camitan and Bajin [16]. If this is the case, resilience must not be necessary for well-being. Also the difference between degree of necessity and degree of sufficiency in the association between well-being and resilience will be evaluated with the extended version of NCA proposed by Sorjonen and Melin [18]. Based on the figures in Camitan and Bajin [16], we hypothesized that analyses with the extended version of NCA would indicate that the association between overall well-being and resilience is characterized by equal degrees of necessity and sufficiency and, consequently, that overall well-being cannot be claimed to be necessary but not sufficient for resilience.

We chose to demonstrate limitations of the original version of NCA with analyses of the association between well-being and resilience because: (1) Camitan and Bajin used NCA to analyze the association between well-being and resilience in a recent study [16]; (2) We believe that Camitan and Bajin’s conclusion, based on analyses with NCA, that well-being is necessary but not sufficient for resilience, may be unwarranted; (3) Camitan and Bajin have made their data publicly available, which allowed us to conduct re-analyses; (4) Research on the association between well-being and resilience is both very interesting and important.

## Method

We refer to the original paper by Camitan and Bajin [16] for a more exhaustive description of the sample, the measures, and data collection procedures. In short, well-being and resilience were measured among 533 Filipino adults (age range 18–40, 29% male) in COVID-19 quarantine. The participants were recruited through social media and data were gathered with an online survey between March 23 and April 10, 2020. Camitan and Bajin used age between 18 and 40, Filipino citizenship, and residence in a quarantined area in the Philippines as inclusion criteria. Of the participants, 35% were college students, 55% were employed, and 10% were unemployed.

Subscales included in the PERMA Profiler, measuring positive emotions (*M* = 7.13, *SD* = 2.03), engagement (*M* = 7.36, *SD* = 1.85), positive relations (*M* = 7.31, *SD* = 2.06), meaning (*M* = 7.27, *SD* = 2.10), accomplishment (*M* = 7.04, *SD* = 1.86), as well as a composite score measuring overall well-being (*M* = 7.27, *SD* = 1.55), were used as measures of well-being. We did not include measures of negative emotions, physical health, and loneliness used by Camitan and Bajin in the present study, as these had no significant necessity effect on resilience (although physical health and loneliness were included in the composite score). Resilience was measured with the Connor-Davidson Resilience Scale (*M* = 24.83, *SD* = 7.22).

In the present study, analyzes were conducted with R 4.1.0 statistical software [22] employing the NCA package [23]. Sufficiency effects of well-being on resilience, operationalized as the size of the empty space in the lower-right corner when plotting variables against each other (see below), were calculated as necessity effects of resilience on well-being (as these two effects are identical). The significance of necessity and sufficiency effects were calculated through 10,000 permutations. Differences between necessity and sufficiency effects were calculated in 10,000 bootstrapped subsamples and the significance of the difference was estimated by calculating a *Z*-score, and then a corresponding *p*-value, by dividing the mean of the differences by the standard error of the differences.

## Results

In Table 1 we see that the well-being variables had a significant necessity effect on resilience. We also see that the sufficiency effect of the five subscale measures of well-being on resilience (= the necessity effect of resilience on these subscales) were non-significant. This might be seen to suggest that well-being was necessary but not sufficient for resilience. However, this conclusion would be compromised by the fact that the difference between the necessity effect and the sufficiency effect was not significant, except for meaning. Moreover, for the overall well-being composite score the sufficiency effect was significant and the difference between the necessity and the sufficiency effect was very non-significant.

**Table 1:** Necessity and sufficiency effects of well-being on resilience, as well as the difference between these two effects

	Necessity		Sufficiency		Difference	
Well-being variable	*d*	*p*	*d*	*p*	*d (SE)*	*p*
Positive Emotions	0.122	< 0.001	0.056	0.226	0.065 (0.035)	0.069
Engagement	0.085	< 0.001	0.026	0.796	0.062 (0.039)	0.109
Positive relations	0.093	< 0.001	0.068	0.088	0.033 (0.019)	0.080
Meaning	0.117	0.003	0.032	0.474	0.088 (0.021)	< 0.001
Accomplishment	0.121	< 0.001	0.085	0.153	0.035 (0.031)	0.264
Overall well-being	0.121	< 0.001	0.137	0.008	0.000 (0.033)	0.991

The sufficiency effect of well-being on resilience equals the necessity effect of resilience on well-being; *d* = effect size; *p* = significance; *SE* Standard error

**Fig. 1:**
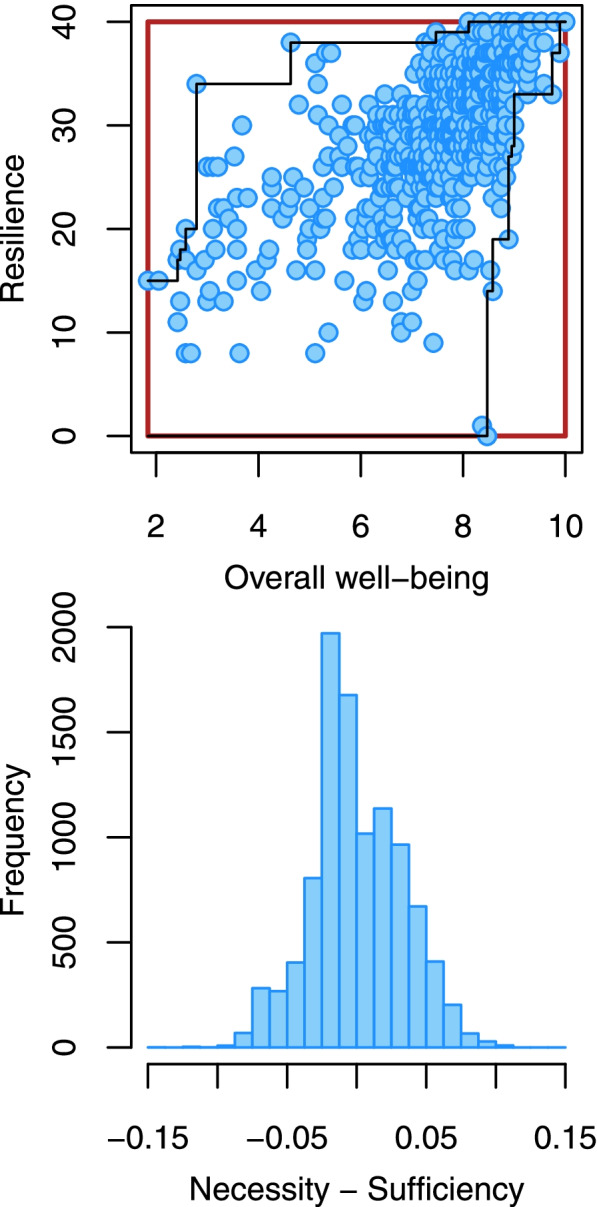
Upper panel: Association between overall well-being and resilience, with ceiling and floor lines indicated. The size of the empty space above/below these lines, as a percentage of the size of the red square, correspond to the necessity and the sufficiency effects, respectively; Bottom panel: Frequency distribution of the differences between the necessity and the sufficiency effect for the association between overall well-being and resilience, calculated in 10,000 bootstrapped subsamples

The necessity and sufficiency effects of the overall well-being composite on resilience, as well as the difference between these two effects, is illustrated in Fig. 1. In the upper panel, the size of the empty space above the ceiling line in the upper-left corner, as a percentage of the red square given by the lowest and highest values on the two variables, corresponds to the necessity effect (d = 0.121) while the size of the empty space below the floor line in the lower-right corner corresponds to the sufficiency effect (d = 0.137) which, as stated above, was identical to the necessity effect of resilience on overall well-being. In the bottom panel we see that the frequency distribution of the differences between the necessity effect and the sufficiency effect, calculated in 10,000 bootstrapped subsamples, firmly included zero, indicating no difference between the two effects.

## Discussion

The present findings suggested that the conclusion by Camitan and Bajin [16], that well-being is necessary but not sufficient for resilience, may be unwarranted. We found that resilience had a significant necessity effect on overall well-being and it would be inconsistent with rules of logic if well-being is necessary but not sufficient for resilience at the same time as resilience is necessary for well-being. Instead, analyses with an extended version of NCA, proposed by Sorjonen and Melin [18], indicated that the association between overall well-being and resilience was characterized by equal degrees of necessity and sufficiency. The present findings could be seen to concur with the apparent uncertainty about which, if any, of well-being and resilience is the antecedent of the other. Some have proposed that positive emotions, e.g. well-being, promote resilience [10, 11], while others have suggested that resilience may facilitate positive emotions [12–15].

We suspect that Camitan and Bajin, and maybe also others, may have been duped by the name of the test as well as the content of Dul’s [1] original paper (including its title) into believing that NCA analyzes if a condition X can be assumed to be necessary, or even necessary but not sufficient, for an outcome Y. However, as later recognized by the developers, NCA is only capable of evaluating if the association between X and Y is characterized by some unspecified type of non-randomness [20]. With this in mind, it might be asked if it would not be appropriate to rename the analysis “a randomness test (ART)” or something similar. Then again, ordinary linear regression analysis seems superior to NCA for identifying non-random associations [21].

### Limitations

The present main finding, that the association between overall well-being and resilience is characterized by equal degrees of necessity and sufficiency, is based on data collected from a Filipino sample in exceptional circumstances (COVID-19 quarantine). It is possible that the association between overall well-being and resilience is characterized by differing degrees of necessity and sufficiency in other samples and circumstances.

## Conclusions

Resilience had a significant necessity effect, as calculated by necessary condition analysis (NCA), on overall well-being. Consequently, well-being does not seem to be a necessary but not sufficient condition for resilience. Instead, analyses with an extended version of NCA suggested that the associations between overall well-being and resilience was characterized by equal degrees of necessity and sufficiency. Some researchers still seem to believe that a significant result in NCA (original version) indicates that the analyzed condition is necessary but not sufficient for the outcome. However, as recognized by the developers, NCA (original version) is only capable of evaluating if the association between two variables is characterized by some unspecified type of non-randomness.

## Data Availability

The empirical data, originally used by Camitan and Bajin [16], is available here (https://www.frontiersin.org/articles/10.3389/fpsyg.2021.558930/full#supplementary-material). The script for the present study is available at the Open Science Framework at https://osf.io/abnj4/.
